# Gleanings from Our Exchanges

**Published:** 1872-02-15

**Authors:** 


### Gleanings from Our Exchanges.

Fungus as a Cause of Whooping-Cough. —The germ theory of disease, which some pathologists seek to extend so widely, has been applied by Dr. Letzerich to explain the extremely infectious disease, whooping-cough. He thinks he has discovered a form of fungoid growth which vegetates in the epithelium of the air-passages, and by its irritation produces the convulsive attacks of coughing. The expectorated mucus in patients suffering from this disease is said to contain masses of brownish-red spores with occasional threads of mycelium, which in later stages of the disease become very abundant. The spores are colored blue by iodine and sulphuric acid. These observations were controlled, first, by cultivation of the spores on pieces of bread soaked in milk, and further, by introducing masses of the fungus growth thus obtained into the tracheæ of young rabbits. This was effected by tracheotomy; but the animals rapidly recovered from the effects of the operation, and in a short time became affected with a cough of a very violent and noisy character—in fact, a genuine whooping-cough. The rabbits thus affected were killed, and their air-passages and lungs found to contain an enormous quantity of the same fungus as that met with in the sputa from human whooping-cough, and, in fact, the mucus expectorated by the rabbits showed precisely the same appearance.—*Half-Yearly Abstract.*

Chloral-Hydrate in Tetanus Monatorum.—Widerhoffer, of Vienna, showed lately to his class a child three months old, which was attacked by *tetanus monatorum* at the end of the first week after birth, and was treated with chloral-hydrate in doses of one and two grains at the time of each onset of convulsions. It was in danger for a fortnight. During the intermissions of the spasms it was fed from the breast by its mother. It is now a fine, healthy-looking child. This is the sixth case (out of ten or twelve) that Dr. Widerhoffer has had recovered under treatment by chloral; under all other methods all his other cases died. Considering that Vogel and other German authorities on children's diseases had until quite recently never seen a case of this affection recover, such a success may be taken to indicate a real advance in therapeutics. Dr. Widerhoffer gives from two to four grain doses of chloral by the rectum, if the infant cannot take it by the mouth.—*Lancet.*

Prepared Collodion for Fractures of the Ribs.—M. Dumas, in the *Montpellier Medical,* summarises in the following manner the rules for the application of this preparation. The substances required, are:—

1. Three pieces of thin muslin of the same breadth from the sternum to the spine, and of a length stretching from the top of the thorax to the second rib below the fracture. If the last rib is fractured, we must make the preparation come down two or three finger breadths on the abdomen, and dispense with reaching the top of the chest. We can, according to the case and the subject, augment the number of muslin, and, of course, of the layers of collodion.

2. Collodion *vicine* as fresh as possible. Mode of application: Apply alternately a coating of collodion and a piece of thin muslin impregnated with this liquid, and reunite the whole by a thick bandage. For that, an assistant is indispensable, for the collodion dries so rapidly that the one who lays on the coatings cannot occupy himself with the *pose* of the muslin.

The author cites two cases, one of which is very curious: the union, retarded by hypertrophy of the heart, had not taken place at the twenty-fifth day. The collodionised preparations assured the formation of callus in a month. In the first case the relief was immediate.—*The Doctor.*

A Lesson for the Day.—The sympathetic influence of the outcries of labor upon pregnant women is well known. In lying-in hospitals it is not unusual for one noisy patient to bring down several others. Even old nurses, long past the climateric, will hold their breath and bear down in sympathy with the expulsive pains. Stock breeders have to guard against this tendency, for sometimes a whole herd will abort after one of their number has dropped her calf.

According to M. Sue (*Essais historiques sur l' Art des Accouch.*, vol. i. p. 599), a midwife, well advanced in pregnancy, while in attendance on a lady of Padua, was herself suddenly seized with the pains of labor. The housemaid, summoned from the basement,—not without many screams, we presume,—pluckily laid down her broom and made shift to deliver mistress and midwife. Unfortunately, she placed the infants—both of the same sex, as bad luck would have it—in the same cradle, where one of them was shortly afterwards found dead. As neither of the women could make out a clear title to the survivor, much wrangling and bad blood resulted.—*Ibid.*

Criminal Abortion.—Ely Van De Warker, M.D., Syracuse, N. Y. (*Jour. Gynæcological Society of Boston*), says that the practitioner ought to bear in mind that instrumental abortion, procured with malicious intent, presents almost always features of malignancy. It is isolated by these features from other accidents of the puerperal state. The innocent abortion is preluded by nature with organic changes which fit the womb for the expulsion of its contents. In the forced abortion demands are made upon a healthy organ for it to instantly violate the laws of its physiological action. This he believes to be the key to the difference between the two cases. —*Med. Record.*

Injection of Alcohol into Cysts.—At the Paris Surgical Society M. Monod lately related some cases in which, after withdrawal of a portion of the fluid from cysts, he injected a little alcohol. The first case was that of a large cystic goitre. One injection greatly diminished the size, and a second, made a fortnight later, completed the cure in a month from the first operation. He was more successful still in hydrocele. The process, he says, does not destroy the tunica vaginalis, but restores it to a normal condition. The alcohol effects a change that enables absorption no longer to be overbalanced by secretion. M. Monad thinks this method of treatment applicable to ovarian and other cysts.—*Ibid.*

Bromide of Iron.—This remedy is advocated by Dr. N. H. Norris, of Beloit, Wis. (*North-Western Med. and Surg. Journal*), as nearly a specific in involuntary seminal emissions and spermatorrhœa. He has administered it three times daily, an hour before or after meals, in doses of from three to five grains, rubbed up in a little syrup.

Prof. Namais (*Practitioner*) states that this remedy corrects defective formation of blood, quiets nervous excitation, and produces the combined effect of iron and the bromides. He regards bromide of iron as being in many instances a therapeutic agent of superior value in epilepsy even to the bromide of potassium. —*Medical Record.*

Ovariotomy Successfully Performed in a Child 6 8-12 Years Old.—Dr. J. Ewing Mears, Philadelphia, (*Phila. Med. Times*), records a successful case of ovariotomy in a child six years and eight months old, which was performed by Dr. W. B. Barker, of Higginsport, Ohio, assisted by Drs. J. J. Bradford and T. S. Bradford, of Augusta, Ky. It is believed to be the earliest age at which the disease is known to have been developed.

Dr. Atlee informs Dr. Mears that in his experience, embracing two hundred and forty-four operations, the youngest age at which he has performed the operation is sixteen years.

Preserving Vaccine Virus.—Dr. D. B. Hillis (*The American Journal of Dental Science;* from *The Pharmacist*) recommends the preservation of vaccine virus between layers of dental red rubber, and says that for several years past he has used no new matter, but has, for the sake of experiment, drawn from the stock originally put up as described. He has always found it effective without paying any regard to temperature. A short time ago he successfully vaccinated a patient from a crust which had been laying about his office twenty-seven months.—*Phil. Medical Times, Dec.* 1, '71.

Fractures of the Femur and Lower Extremity.—Dr. Stromeyer (*British Medical Journal*), in his ambulance at Floing, close by Sedan, where a good deal of hard fighting took place, treated then thirty-four fractures of the femur, and in twenty-four there was a prospect of cure. Four were doubtful cases, and only six died. His table of results in fractures of the leg is also very satisfactory. out of thirty-one fractures of tibia and fibula, or both, caused by gunshot injury, only three died, while the result was doubtful in six instances.—*Medical Record.*

The Prophylaxis of Vaccination.—The protective influence of vaccination is illustrated nowhere more conclusively than by the following facts:

Before the year 1829, according to M. Levy, the ratio of deaths from variola and varioloid in the French Army per 1000 deaths from all causes was 39. After new instructions by the minister of war requiring general revaccination the proportion of deaths fell to 17.5 per 1000 deaths from all causes.—*Bartholow's Jewett Prize Essay*, 1869, *p.* 59.

Excision of the Knee for Disease.—According to John H. Parker, M. D., Surgeon to the Episcopal Hospital, Philadelphia, (*Phila. Med. Times*) seven excisions of the knee have been performed within the past two years in the city of Philadelphia. They were all, like his own two cases, satisfactory so far as the local treatment and its effects were concerned. He regards this procedure as one deserving of further trial at the hands of American surgeons, although, heretofore, it has found less favor in this country than abroad.—*Medical Record.*

Whooping-cough and Cod Liver Oil.—Several cases of whooping-cough that came under the professional care of Mr. J. Prestwich, Oldham, have been treated by the administration of cod-liver oil, in drachm or two drachm doses. Mr. Prestwich states that, in the three specimen cases submitted, improvement was marked and recovery unretarded, although in one of these cases the little patient had suffered for nine months prior to the cod-liver oil treatment.—*The Doctor.*

Traumatic Tetanus Cured with Hydrate of Chloral.—J. Suydam Knox, M.D., of Somerville, New Jersey (*Am. Jour. Med. Sciences*), relates the case of a boy æt. 14, with traumatic tetanus, which was cured by hydrate of chloral, in 30-grain doses, taken every four hours in syr. acaciæ. The chloral promptly controlled the spasms and induced sleep. At the end of four days it was administered only night and morning.

The Fate of Indian Doctors.—The practice of medicine among the Indian nations leads to as unpleasant consequences as those we recently referred to as having happened to a physician in Egypt. Indian Joe, a Piute medicine-man, well known among the whites, having failed to restore to health two sick Indians, was stoned to death by his tribe.—*Medical Times.*

